# Transcriptional regulation of the IL-13Rα2 gene in human lung fibroblasts

**DOI:** 10.1038/s41598-020-57972-1

**Published:** 2020-01-23

**Authors:** Loka R. Penke, Hideyasu Ouchi, Jennifer M. Speth, Njira Lugogo, Yvonne J. Huang, Steven K. Huang, Marc Peters-Golden

**Affiliations:** 1 Division of Pulmonary and Critical Care Medicine, Department of Internal Medicine, University of Michigan Medical School, Ann Arbor, MI USA; 2 Graduate Program in Immunology, University of Michigan Medical School, Ann Arbor, MI USA

**Keywords:** Transcriptional regulatory elements, Interleukins, Growth factor signalling, Lipid signalling

## Abstract

Interleukin (IL)−13 is a type 2 cytokine with important roles in allergic diseases, asthma, and tissue fibrosis. Its receptor (R) α1 is primarily responsible for the biological actions of this cytokine, while Rα2 possesses a decoy function which can block IL-13 signaling. Although the expression of Rα2 is known to be subject to modulation, information about its transcriptional regulation is limited. In this study, we sought to expand the understanding of transcriptional control of Rα2 in lung fibroblasts. We confirmed previous reports that IL-13 elicited modest induction of Rα2 in normal adult human lung fibroblasts, but found that prostaglandin E_2_ (PGE_2_) and fibroblast growth factor 2 (FGF-2) –mediators known to influence fibroblast activation in tissue fibrosis but not previously investigated in this regard – led to a much greater magnitude of Rα2 induction. Although both PGE_2_ (via protein kinase A) and FGF-2 (via protein kinase B, also known as AKT) depended on activation of cAMP-responsive element-binding protein (CREB) for induction of Rα2 expression, they nevertheless demonstrated synergy in doing so, likely attributable to their differential utilization of distinct transcriptional start sites on the Rα2 promoter. Our data identify CREB activation via PGE_2_ and FGF-2 as a previously unrecognized molecular controller of Rα2 gene induction and provide potential new insights into strategies for therapeutic manipulation of this endogenous brake on IL-13 signaling.

## Introduction

Interleukin (IL)−13 is an important type 2 cytokine best known for its roles in allergic diseases and asthma^1^, but which also contributes to other inflammatory diseases such as Crohn’s disease and ulcerative colitis^2^, tissue fibrosis^3,4^, and various forms of cancer^5–7^. IL-13 recognition is complex, involving heterodimers comprised of three distinct receptor subunits. IL-13 receptor α1 (IL-13Rα1 or Rα1) has low affinity for IL-13, but when associated with the IL-4 receptor (IL-4Rα) subunit, ligand binding results in productive signaling via JAK/STAT6^8,9^. IL-13 receptor α2 (IL-13Rα2 or Rα2) has greater affinity for IL-13, but its impact on signaling upon ligand binding depends on cell type and context^10,11^. Rα2 lacks a cytoplasmic domain, and accordingly, many studies have reported that Rα2 fails to initiate signal transduction^10^ and in fact, can act as a decoy receptor capable of binding ligand and thus preventing productive signaling through Rα1^12–15^. On the other hand, more recent reports have identified cytoplasmic interactors for Rα2^16^ and described Rα2-mediated activation of various intracellular signaling pathways^17–19^. In keeping with such divergent signaling responses, Rα2 has been reported to both mediate and attenuate mouse models of allergic asthma^20–22^ as well as pulmonary fibrosis^18,23,24^.

Although Rα1 is exclusively a membrane-bound receptor, mouse Rα2 has been shown to exist in membrane-bound as well as soluble forms, which reflect distinct splice variants^25,26^. Although the soluble form does not exist in humans, release of soluble Rα2 through matrix metalloproteinase 8-mediated cleavage of membrane receptor has been demonstrated in both mouse and human cells^27^. Of note, both of these soluble forms of Rα2 have ~ 3-fold greater affinity for IL-13 than does the membrane-bound form, and are capable of inhibiting signaling responses^26–28^. In human lung fibroblasts (Fibs), both membrane and soluble forms of Rα2 have been reported to inhibit the IL-13-induced expression of fibrotic genes^24^.

While expression of Rα1 is ubiquitous in most normal tissues^29–31^, it is not recognized to be subject to substantial modulation. By contrast, while the basal expression of Rα2 in normal tissues is more restricted^32^, its expression is subject to dynamic regulation. For example, Rα2 expression has been reported to be high in a variety of neoplasms^33–36^ as well as in lung Fibs derived from patients with the serious fibrotic lung disease, idiopathic pulmonary fibrosis (IPF)^37,38^. On the other hand, its expression was lower in airway Fibs from patients with asthma than from non-asthmatics^39^. Expression of Rα2 has also been shown to be potentiated by a variety of inflammatory mediators, including IL-13 itself, IL-4, TNF-α, and lysophosphatidic acid (LPA)^40–42^. Of these, only induction by IL-13 has been mechanistically characterized, and – unsurprisingly – shown to proceed via STAT6 activation^43^.

Although Rα2 expression is subject to dynamic control and to dysregulation in a variety of pathological conditions, information about the factors and underlying molecular mechanisms that control transcription of this gene remains quite limited. Understanding the control of its expression is clearly relevant regardless of whether Rα2 serves to mediate or to quench IL-13 signaling. In this study, we have used human lung Fibs to characterize the effects of prostaglandin E_2_ (PGE_2_) and fibroblast growth factor (FGF)−2 on gene expression of Rα2. Although these pleiotropic mediators are implicated in numerous biological processes including inflammation, fibrosis, and cancer, they have not previously been studied in the context of Rα2 expression. We report herein that these substances elicit a far greater magnitude of Rα2 mRNA induction than does IL-13, and we identify a STAT-independent Rα2 transcription mechanism by which they act.

## Results

### Lung Fibs, but not lung epithelial cells or alveolar macrophages, constitutively express Rα2

**Figure 1:**
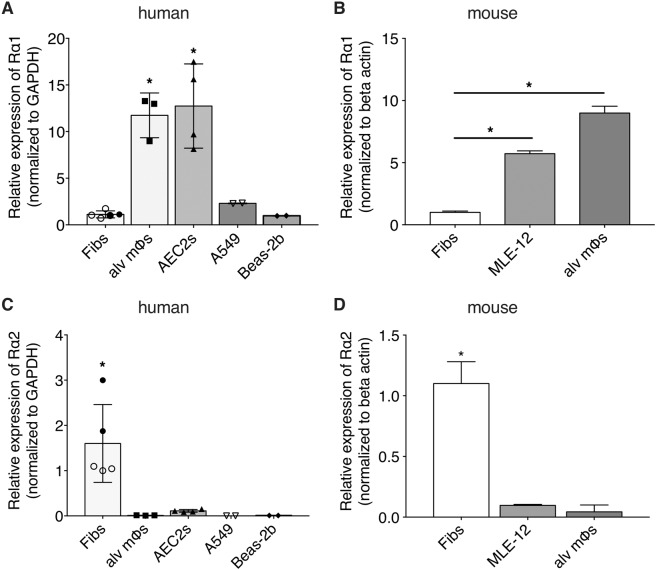
Basal expression of Rα1 and Rα2 in various lung cell types. qPCR analysis of basal expression of Rα1 (**A,B**) and Rα2 (**C,D**) in primary human lung cells (Fibs, alv mϕs, AEC2s Beas-2b and A549 (**A,C**) and in mouse primary lung Fibs, MLE-12 lung epithelial cells, and primary alveolar mϕs (**B,D**). Expression is relative to that of CCL210 Fibs, represented as open circles. Each bar represents mean values (±S.E.) from three independent samples. *p < 0.05.

The relative mRNA expression of Rα1 and Rα2 was determined in primary adult normal human lung Fibs from two distinct sources (those isolated at our institution from the lungs of two subjects, and commercially available CCL210 cells). We also studied primary human alveolar macrophages [alv mϕs] and primary human type II alveolar epithelial cells [AEC2s]) isolated at our institution, and human lung epithelial cell lines obtained commercially (Beas-2b and A549). As shown in Fig. 1A, all three human lung cell types exhibited readily demonstrable baseline expression of Rα1. This was also evident in primary lung Fibs, a lung epithelial cell line (MLE-12), and primary alv mϕs derived from mouse (Fig. 1B). In both human and mouse, both epithelial cells and alv mϕs manifested higher levels of Rα1 mRNA than did Fibs from the lung. Conversely, Rα2 transcript was expressed only in lung Fibs, but not in lung epithelial cells or alv mϕs from either humans (Fig. 1C) or mouse (Fig. 1D). Our finding that among lung cells, basal expression of Rα2 is restricted to Fibs is consistent with single-cell transcriptomic studies reported in human^44^ and mouse^45^ lung tissues. Delta Ct values for human Rα1 and Rα2 (normalized to GAPDH) are provided in Supplementary Table 1.

### Upregulation of Rα2 expression in lung Fibs by soluble mediators

**Figure 2:**
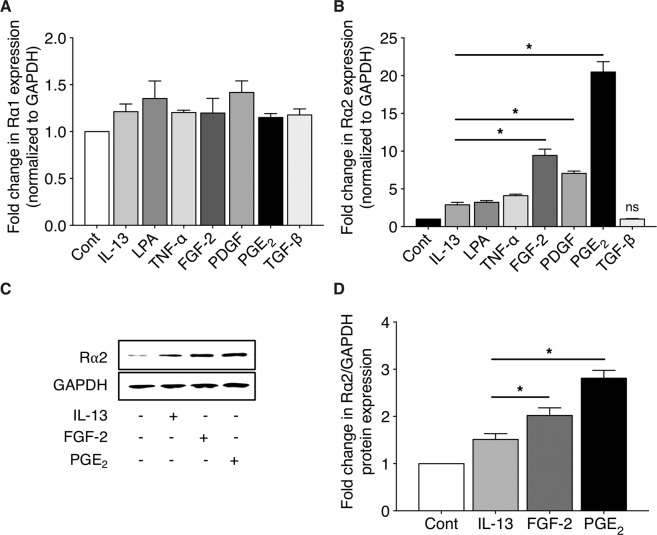
Stimulated expression of Rα1 and Rα2 in human lung Fibs. CCL210 cells were stimulated with IL-13, TNF-α, FGF-2, PDGF, PGE_2_, or TGF-β for 24 h. Expression of Rα1 (**A**) and Rα2 (**B**) were determined using qPCR analysis. In (**A)**, none of the values are significantly different from untreated control. In (**B**), all treatments except TGF-β are significantly greater than control (p < 0.05); the asterisks indicate values significantly greater than IL-13. (**C-D**) CCL210 cells were stimulated with IL-13, FGF-2, or PGE_2_ for 48 h and expression of Rα2 protein was determined by western blot; the blot shown in (**C**) is representative of 3 independent experiments, and quantification of western blots by mean densitometric analysis is shown in (**D**). Each bar represents mean values (±S.E.) from three independent experiments. In (**D**), all values are significantly greater than control (p < 0.05); asterisks indicate values significantly greater than IL-13. *p < 0.05; ns, not significant.

Next, we assessed the influence of a variety of soluble mediators with effects on inflammation and fibrogenesis on Rα1 and Rα2 expression in CCL210 Fibs. As shown in Fig. 2A, treatment with IL-13, TNF-α, FGF-2, PDGF, PGE_2_ and TGF-β failed to alter basal Rα1 mRNA expression. In contrast, and consistent with literature reports, Rα2 mRNA expression was modestly but significantly upregulated by IL-13, LPA, and TNF-α (Fig. 2B). Rα2 expression was also significantly upregulated by FGF-2, PDGF, and PGE_2_, but not by TGF-β. Of note, the fold induction of Rα2 by FGF-2 and PGE_2_ (~10- to 20-fold) was markedly higher than that elicited by IL-13, LPA, and TNF-α (~2- to 4-fold). The doses of PGE_2_ and FGF-2 utilized were previously established as optimal for other actions in human lung Fibs^46^. Kinetic analysis of Rα2 induction by FGF-2 and PGE_2_ in CCL210 lung Fibs showed its onset at 3–6 h and a plateau reached at 36–48 h after addition (Supplementary Figure 1A). In contrast to their actions in Fibs, neither FGF-2, PDGF, nor PGE_2_ showed any effect on Rα2 expression in either A549 lung epithelial cells or primary human alv mϕs (Supplementary Figure 1B). Although an increase in expression of intracellular Rα2 protein in Fibs by FGF-2 and particularly PGE_2_ (Fig. 2C,D) accompanied that of mRNA (Fig. 2B), the magnitude of induction was substantially less. This prompted us to evaluate the presence of the cleaved and secreted form of Rα2 in Fib conditioned medium^27^. A modest increase in Rα2 protein in Fib culture supernatant was noted in response to FGF-2 and PGE_2_ (Supplementary Figure 1C). To verify the functional effects of upregulated expression of Rα2 on IL-13 signaling, we examined expression of the important Rα1 target gene periostin. As proof-of-concept, transfection with a CMV promoter-driven Rα2 construct dampened baseline and IL-13-induced periostin gene expression (Supplementary Figure 2A). Likewise, pre-treatment of cells with FGF-2 or PGE_2_ also dampened baseline and IL-13-induced levels of this matricellular gene (Supplementary Figure 2B). Co-stimulation with PGE_2_ + FGF-2 also markedly attenuated IL-13-induced periostin gene expression; however, this reduction was not significantly greater than that seen when cells were pretreated with PGE_2_ prior to IL-13 stimulation. Taken together, these data clearly show that induction of Rα2 transcript and protein in response to FGF-2 and PGE_2_ functionally dampens IL-13-induced signaling via Rα1 in lung Fibs.

### Induction of Rα2 expression in response to PGE_2_ and FGF-2 requires new transcription

**Figure 3:**
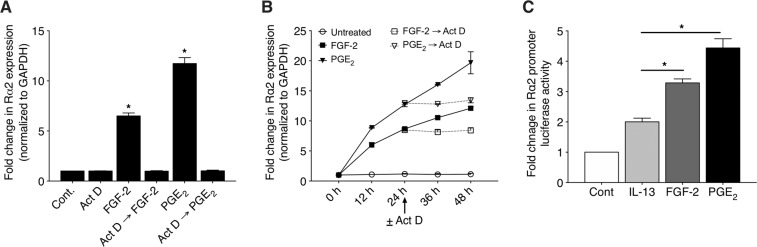
FGF-2 and PGE_2_ increase Rα2 expression via new transcription. (**A**) CCL210 cells were pre-treated ± Act D for 30 min followed by stimulation ± FGF-2 or ± PGE_2_ for 24 h, and the expression of Rα2 was determined using qPCR analysis. (**B**) CCL210 cells were stimulated ± FGF-2 or ± PGE_2_ for 24 h and then treated ± Act D. Samples were harvested at 0 h, 12 h, 24 h (prior to Act D treatment) as well as at 36 h and 48 h, and the expression of Rα2 was determined by qPCR analysis. (**C**) CCL210 cells were transfected with Rα2 promoter luciferase construct (pGL3- Rα2), stimulated with IL-13, FGF-2 or PGE_2_ for 24 h, and luciferase activity determined using Dual-Luciferase reporter assay system. Each bar represents mean values (±S.E.) from three independent experiments. All stimulated values were greater than control (p < 0.05). The asterisks depicted in C indicate values significantly greater than IL-13. *p < 0.05.

We next sought to determine the relative roles of new transcription versus increased stability in the increase in Rα2 mRNA in PGE_2_- and FGF-2-stimulated Fibs. As shown in Fig. 3A, pre-treatment with the transcription inhibitor actinomycin D (Act D) did not affect the baseline expression of Rα2, but completely prevented the ability of both PGE_2_ and FGF-2 to increase Rα2 transcripts. Furthermore, the addition of Act D 24 h after initial treatment with PGE_2_ and FGF-2 stopped further transcript accumulation but failed to reveal any attenuation of mRNA decay (Fig. 3B). These data suggest that increases in Rα2 mRNA accumulation by PGE_2_ and FGF-2 reflect increased transcription rather than decreased degradation. To directly assess the induction of Rα2 transcription by PGE_2_ and FGF-2 at the promoter level, and to compare their actions to the positive control, IL-13^47^, CCL210 cells were transfected with a Rα2 promoter luciferase construct (pGL3-Rα2). As shown in Fig. 3C, and consistent with the Rα2 mRNA data shown in Figs. 2 and 3A, stimulation with either PGE_2_ or FGF-2 increased the Rα2 promoter activity and did so to a greater extent than did IL-13.

### PGE_2_-induced expression of Rα2 proceeds via an EP2/cAMP/PKA pathway

**Figure 4:**
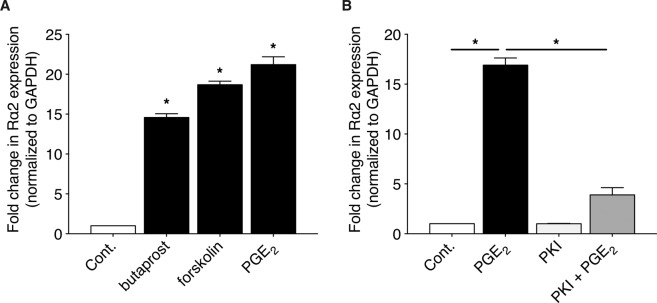
PGE_2_ increases Rα2 expression via an EP2/cAMP/PKA pathway. (**A**) CCL210 cells were treated with PGE_2_, butaprost, or forskolin for 24 h and the expression of Rα2 was determined using qPCR analysis. (**B**) CCL210 cells were pre-treated ± PKI for 30 min followed by stimulation ± PGE_2_ for 24 h, and expression of Rα2 was determined using qPCR analysis. Each bar represents mean values (±S.E.) from three independent experiments. *p < 0.05.

PGE_2_ can ligate four different G protein-coupled receptors (EP1–4) activating distinct signaling pathways. We previously reported that PGE_2_ signaling in CCL210 cells is mainly through an EP2 receptor-mediated increase in cAMP signaling^48^. The role of EP2 and cAMP signaling in Rα2 induction by PGE_2_ was therefore assessed. As shown in Fig. 4A, Rα2 expression was upregulated by butaprost (an analog of PGE_2_ that is a selective agonist for EP2) and forskolin (a direct activator of adenylyl cyclase that generates cAMP) in a manner parallel to that of PGE_2_. We also assessed the role of the classical cAMP effector PKA in PGE_2_-induced Rα2 induction. As shown in Fig. 4B, inhibition of PKA by a myristoylated and thus cell permeable PKA inhibitor, PKI 14–22 amide, completely abolished the ability of PGE_2_ to induce the expression of Rα2. Together, these data indicate that PGE_2_ utilizes an EP2/cAMP/PKA pathway to induce transcription of Rα2.

### FGF-2-induced expression of Rα2 proceeds via a PI3 kinase/PDK1/AKT pathway

**Figure 5:**
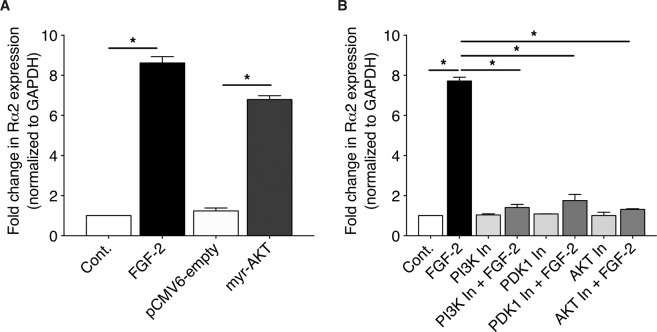
FGF-2 increases Rα2 expression via a PI3 kinase/PDK1/AKT pathway. (**A**) CCL210 cells were treated with FGF-2 or transfected with pCMV6 (empty) vector or myr-AKT vector for 24 h, and the expression of Rα2 was determined using qPCR analysis. (**B**) CCL210 cells were pre-treated ± PI3 kinase inhibitor (PI3K In), PDK1 In, or AKT In for 30 min followed by stimulation ± FGF-2 for 24 h, and the expression of Rα2 was determined using qPCR analysis. Each bar represents mean values ( ± S.E.) from three independent experiments. *p < 0.05.

We previously reported that in human lung Fibs, signaling by mitogens such as FGF-2 critically depends on activation of the phosphoinositide-3-kinase (PI3 kinase)/3-phosphoinositide-dependent protein kinase 1 (PDK1)/AKT pathway^46^. We therefore interrogated the involvement of this pathway in the induction of Rα2 by FGF-2. First, we verified the sufficiency of this molecular pathway for the induction of Rα2 in Fibs by transfecting cells with a constitutively active form of AKT (myr-AKT). As shown in Fig. 5A, increased expression of Rα2 was observed in CCL210 cells expressing myr-AKT, but not empty vector. Next, we utilized a variety of pharmacologic inhibitors to determine the necessity of this pathway in FGF-2-stimulated cells. As shown in Fig. 5B, pre-treatment of CCL210 cells with three distinct PI3 kinase pathway-specific inhibitors (LY294002, inhibitor of PI3 kinase; GSK 2334470, inhibitor of PDK1; and triciribine, inhibitor of AKT) markedly reduced FGF-2-induced expression of Rα2.

### PGE_2_ and FGF-2 demonstrate synergy in the induction of Rα2 gene expression

**Figure 6:**
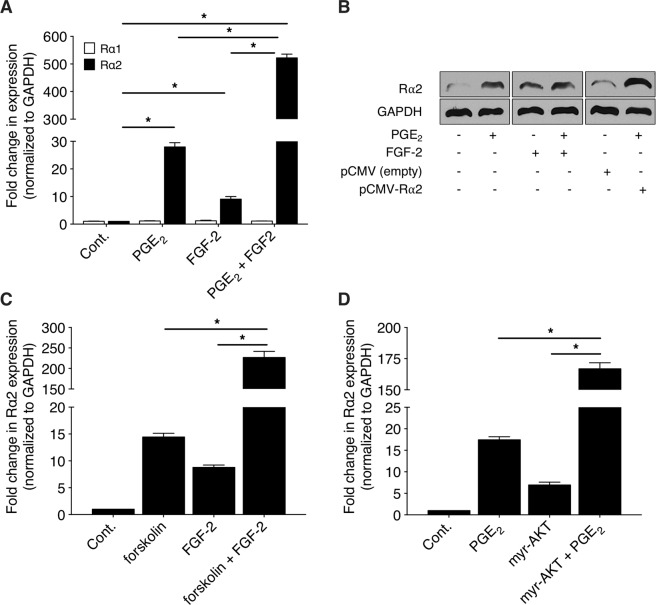
Induction of Rα2 by PGE_2_/forskolin plus FGF-2/myr-AKT is synergistic. (**A-B**) CCL210 cells were stimulated with PGE_2_, FGF-2 or both for 24 h and 48 h and the expression of Rα1 and Rα2 were determined using qPCR analysis (**A**) and western blot (**B**), respectively. (**B**) also depicts protein expression in cells over-expressing Rα2 (mean induction of 6-fold over empty vector). Blot in (**B**) is representative of 3 independent experiments. (**C**) CCL210 cells were stimulated with forskolin, FGF-2, or both for 24 h and the expression of Rα2 was determined using qPCR analysis. (**D**) CCL210 cells were stimulated with PGE_2_, transfected with myr-AKT, or both for 24 h and the expression of Rα2 was determined using qPCR analysis. In A, C and D, all treatments are significantly greater than control (p < 0.05). Each bar in A, C and D represents mean values ( ± S.E.) from three independent experiments.

The fact that PGE_2_ and FGF-2 both stimulate Rα2 expression was somewhat unexpected, as we and others have reported that PGE_2_ acts as a negative regulator of a number of FGF-2-induced actions in lung Fibs, including proliferation, migration and survival^46,49^. It was therefore of interest to examine the effects of co-stimulation with both mediators on Rα2 expression. As shown in Fig. 6A, co-stimulation of CCL210 cells with PGE_2_ and FGF-2 resulted in a markedly synergistic increase of several hundred-fold in Rα2 mRNA expression. Consistent with the findings shown in Fig. 2A with individual mediators, co-stimulation with PGE_2_ and FGF-2 likewise failed to increase Rα1 expression. Although a synergistic effect was also present at the protein level, it was, as also demonstrated for these agonists individually (as shown in Fig. 2), much less striking than for mRNA (Fig. 6B). As shown in Fig. 6B, even CMV promoter-driven Rα2 overexpression resulted in no higher level of Rα2 protein induction than that observed with PGE_2_ + FGF-2 treatment. This suggests either that the capacity for translation of Rα2 mRNA is saturated, or that additional post-transcriptional levels of regulation influence Rα2 protein induction. Co-stimulation with forskolin (instead of PGE_2_) and FGF-2 also resulted in a synergistic degree of Rα2 induction (Fig. 6C). Likewise, stimulation of CCL210 cells expressing myr-AKT (instead of treatment with FGF-2) with PGE_2_ also resulted in synergy (Fig. 6D).

### CREB activation is necessary for both PGE_2_- and FGF-2-induced expression of Rα2

**Figure 7:**
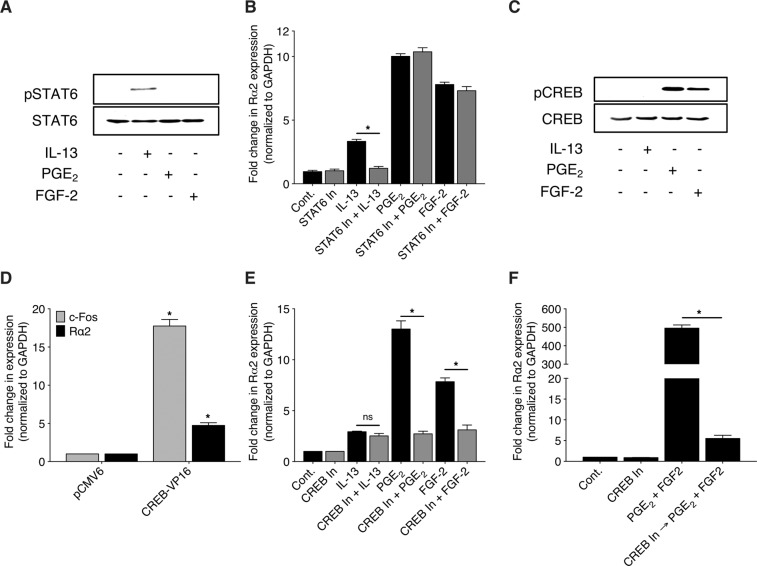
Rα2 induction by IL-13 is mediated by STAT6 while induction by FGF-2 and PGE_2_ is mediated by CREB. (**A**) CCL210 cells were stimulated with either IL-13, PGE_2_, or FGF-2 for 10 min and total and phosphorylated STAT6 were determined by western blot; blot shown is representative of 3 independent experiments. (**B**) CCL210 cells were pre-treated ± STAT6 In for 30 min followed by stimulation ± IL-13, PGE_2_ or FGF-2 for 24 h, and the expression of Rα2 was determined using qPCR analysis. (**C**) CCL210 cells were stimulated with either IL-13, PGE_2_, or FGF-2 for 10 min and total and phosphorylated CREB were determined by western blot; blot shown is representative of 3 independent experiments. (**D**) CCL210 cells were transfected with pCMV6 (empty) vector or CREB-VP16 construct for 24 h and the expression of prototypical CREB target gene c-Fos as well as Rα2 were determined using qPCR analysis. (**E**) CCL210 cells were pre-treated ± CREB In for 30 min followed by stimulation ± IL-13, ± PGE_2_ or ± FGF-2 for 24 h and the expression of Rα2 was determined using qPCR analysis. (**F**) CCL210 cells were pre-treated ± CREB In for 30 min followed by co-stimulation ± PGE_2_ plus FGF-2 for 24 h, and the expression of Rα2 was determined using qPCR analysis. Each bar in B, D, E and F represents mean values ( ± S.E.) from three independent experiments. *p < 0.05; ns, not significant.

Since phosphorylation and activation of STAT6 has been implicated in IL-13-induced Rα2 expression, we assessed the activation of STAT6 by PGE_2_ and FGF-2. Unlike IL-13, PGE_2_ and FGF-2 failed to elicit phosphorylation of STAT6 (Fig. 7A). Likewise, pharmacologic inhibition of STAT6 using AS1517499 abolished IL-13-, but not PGE_2_- or FGF-2-induced Rα2 expression (Fig. 7B). CREB has long been recognized as an important transcriptional effector downstream of PGE_2_/cAMP/PKA^50,51^, and phosphorylation and activation of CREB have more recently also been documented to participate in FGF-2/AKT signaling^52,53^. Although CREB has not, to our knowledge, previously been investigated in the transcriptional control of Rα2, it is noteworthy that MatInspector (http://www.genomatix.de/matinspector.html) identified several CREB-binding sites, in addition to STAT binding sites, within the Rα2 promoter (Supplementary Figure 3). We therefore evaluated the role of CREB in Rα2 induction. Consistent with this possibility, both PGE_2_ and FGF-2 increased the phosphorylation of CREB, while IL-13 had no such effect (Fig. 7C). As shown in Fig. 7D, forced overexpression of a constitutively active form of CREB (CREB-VP16) resulted in a marked increase in the expression of the well-known CREB target gene c-Fos and a more modest increase in that of Rα2. To assess the necessity of CREB activation in PGE_2_- and FGF-2-mediated Rα2 expression, we employed the potent and selective cell-permeable CREB inhibitor 666–15. As shown in Fig. 7E, pre-treatment with 666–15 abrogated the induction of Rα2 by both PGE_2_ and FGF-2. Pharmacologic inhibition of CREB activation also abrogated the marked synergistic effect of PGE_2_ and FGF-2 on Rα2 induction (Fig. 7F).

### PGE_2_- and FGF-2-mediated upregulation of Rα2 expression involves transcriptional activation from distinct start sites

**Figure 8:**
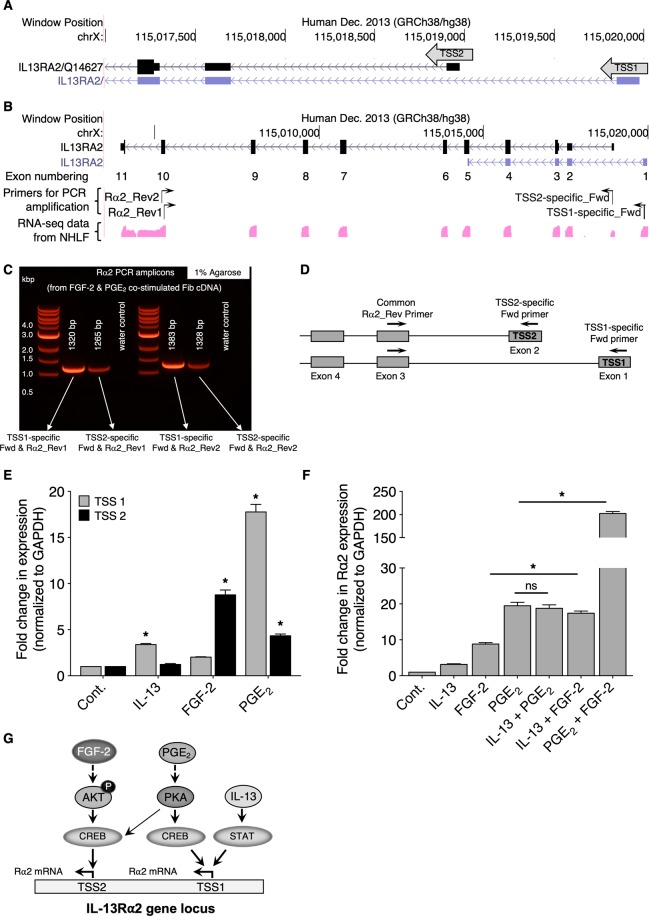
Transcriptional activation of Rα2 by various stimuli at two distinct transcription start sites. (**A**) Depiction of TSS1 and TSS2 locations for Rα2 (based on UCSC database). (**B**) Depiction of exonic positions on Rα2 gene (based on ENCODE RNA-seq data from normal human lung Fibs), and representation of the design of TSS-specific forward primers and a pair of reverse primers (Rα2_Rev1 and Rα2_Rev2) both bind to exon 10 but at different locations. (**C**) Agarose gel image showing TSS1- and TSS2-specific PCR amplicons and their product size. (**D**) Schematic representation of the design of TSS-specific qPCR primers with a common reverse primer, Rα2_Rev Primer1 to distinguish distinct TSSs. (**E**) CCL210 cells were stimulated with IL-13, FGF-2, or PGE_2_ for 24 h and the expression of distinct Rα2 transcripts were assessed by qPCR using TSS1-specific and TSS2-speicfic primers. (**F**) CCL210 cells were stimulated with IL-13, FGF-2, PGE_2_, IL-13 + FGF-2, IL-13 + PGE_2_, or FGF-2 + PGE_2_ for 24 h and the expression of Rα2 was determined using qPCR analysis with the common primer. Each bar in C and D represents mean values ( ± S.E.) from three independent experiments. In C, the asterisks indicate treatments are significantly greater than control (p < 0.05). In D, all treatments are significantly greater than control (p < 0.05); the asterisks indicate values significantly greater than IL-13. (**G**) Summary scheme demonstrating the signaling pathways, transcription factors, and TSSs through which IL-13, PGE_2_, and FGF-2 act to induce Rα2 transcription.

Normal human lung Fib RNA-seq data available at the UCSC genome browser (https://genome.ucsc.edu/) reveal the existence of two distinct transcriptional start sites (TSSs) for Rα2 (Fig. 8A). We wished to determine the TSS utilized by IL-13 and to interrogate the site(s) utilized by PGE_2_ and FGF-2. Therefore, we arbitrarily designated the two distinct TSSs as TSS1 and TSS2. The UCSC genome browser depicts a partial sequence for the TSS1-initiated transcript, and additional data available at the Ensembl browser (http://useast.ensembl.org/Homo_sapiens/Location/View?db=core;g=ENSG00000123496;r=X:115003655-115020297) suggest that the annotated partial transcript from TSS1 represents a long non-coding RNA. In contrast, our data suggest that both IL-13 and PGE_2_ induce a TSS1-initiated transcript and make Rα2 protein (as shown in Fig. 2C). Moreover, RNA-seq from normal human lung Fibs shows expression of full-length transcript from TSS1 (see Supplementary Figure 4). Therefore, we attempted to characterize the TSS1-initiated transcript by PCR amplification followed by DNA sequencing. As shown in Fig. 8B, using TSS-specific forward primers and a pair of reverse primers (Rα2_Rev1 and Rα2_Rev2 that bind to exon 10 but at different regions (see Supplementary Table 2B), we PCR-amplified the Rα2 transcript. Since FGF-2 and PGE_2_ show synergistic effects on Rα2 mRNA expression (Fig. 6A), we utilized cDNA from Fibs co-stimulated with FGF-2 and PGE_2_. As shown in Fig. 8B, amplification of the Rα2 transcript from both TSS1- and TSS2-specific primers was determined by separation in 1% agarose gel yielding PCR amplicons at the expected length (Fig. 8C). Next, DNA sequencing was performed on the PCR amplicons following their purification from agarose gel (see Supplementary Table 3), which confirmed the expression of full-length Rα2 transcripts initiated by both TSS1 and TSS2 in human lung Fibs.

To explore the TSS utilization by IL-13, PGE_2_, and FGF-2, as shown in Fig. 8D, we generated TSS-specific qPCR primer sets (see sequences in Supplementary Table 2B). To ensure that the TSS1-initiated transcript did not include TSS2, we utilized a different reverse primer, Rev primer2 (see Supplementary Table 2C) along with Fwd Primer1 and Fwd Primer2 and amplified the TSS1 and 2 products, respectively. As shown in Supplementary Figure 5, an SspI restriction site (AAT/ATT) is available within the TSS2 sequence, and digestion with SspI enzyme resulted in fragmentation of the TSS2 PCR product but not the TSS1 PCR product. Next, as shown in Fig. 8E, IL-13 utilized TSS1 exclusively. PGE_2_ also preferentially utilized TSS1 to initiate Rα2 transcription, whereas FGF-2 preferentially utilized TSS2. As predicted by this pattern of TSS utilization, co-stimulation with IL-13 plus PGE_2_ (both acting via TSS1) yielded no synergy as compared to either alone, while co-stimulation with IL-13 (acting via TSS1) and FGF-2 (acting via TSS2) demonstrated synergy in Rα2 expression as compared to that observed with either stimulus alone (Fig. 8F). These data indicate that differential utilization of these two TSSs by various agonists may explain interactions in transcription of Rα2. Figure 8G summarizes our findings of differential utilization of distinct Rα2 TSSs by IL-13/STAT6, PGE_2_/EP2/cAMP/PKA/CREB, and FGF-2/PI3 kinase/PDK1/AKT/CREB pathways.

## Discussion

IL-13 is a pleiotropic type 2 cytokine with well-recognized roles in inflammatory, immune, fibrotic, and neoplastic processes. Its receptors Rα1 and Rα2 differ in a number of respects. From a functional perspective, while Rα1 is well known to exert biological actions via JAK/STAT6 signaling, Rα2 in most experimental systems – including lung Fibs^54–57^ – acts as a decoy receptor dampening the actions of IL-13. Another key difference is that unlike Rα1, expression of Rα2 has been shown to be subject to modulation in various disease states and in response to various mediators. Despite this, knowledge about the molecular regulation of Rα2 gene expression is quite limited. In this study, we found that Rα2 gene transcription in human lung Fibs was strongly induced by PGE_2_, FGF-2, and PDGF – mediators known to exhibit diverse regulatory effects on Fibs. Such induction of Rα2 was not seen in lung epithelial cells or alv mϕs. Although the effects of PGE_2_ and FGF-2 proceeded via distinct and separate signaling pathways, their actions converged on CREB – the first time this transcription factor has been implicated in the control of Rα2 gene transcription.

The UCSC genome browser identifies at least two distinct TSSs in the human Rα2 gene and ENCODE RNA-seq analysis in normal human lung Fibs further confirmed the expression of Rα2 transcripts from both of these TSSs. However, only a partial sequence was deposited at the UCSC database, and it was annotated, based on computational prediction by Havana (Human and Vertebrate Analysis and Annotation), as a lncRNA 218. By contrast, our experimental data clearly demonstrate full-length Rα2 transcript initiated from TSS1. Finally, we identified that synergistic interactions among mediators in Rα2 induction are associated with CREB-mediated transcription initiation at distinct TSSs. Together, these data expand our understanding of the mechanisms governing Rα2 gene transcription.

The actions of IL-13 have been well studied in a variety of lung cell types, including epithelial cells, mϕs, eosinophils, Fibs, and dendritic cells^58–61^. However, the relative expression of its Rα1 and Rα2 subunits among key lung cells has never been reported. We found high basal expression of Rα1 in lung epithelial cells, mϕs, and Fibs, consistent with IL-13 responses in these cell types being Rα1-dependent. By contrast, Rα2 was basally expressed only in Fibs and was undetectable in epithelial cells and alv mϕs. However, as reported previously, its expression in epithelial cells can be induced by IL-13 to function as a feedback inhibitory loop in these cells^47^. Basal expression of Rα2 in lung Fibs was modestly enhanced by IL-13 stimulation, as reported previously^62^. We also verified previous reports that LPA and TNF-α modestly upregulated Rα2 expression. The effects of mitogenic factors FGF-2 and PDGF on Fib Rα2 had not previously been reported, and we found that these markedly increased the expression of Rα2 in Fibs. However, the well-characterized pro-fibrotic factor TGF-β showed no effect on Rα2. These data suggest a potential role for fibrotic drivers other than TGF-β in Rα2 upregulation in lung Fibs during lung fibrosis. Unexpectedly, PGE_2_, a well-known suppressor of numerous Fib processes including proliferation, migration, and differentiation and thereby fibrosis, also strongly enhanced the expression of Rα2 in Fibs.

Kinetic studies with Act D as well as Rα2 promoter luciferase assays revealed that both PGE_2_ and FGF-2 increased expression of the Rα2 gene by enhancing transcription, rather than by impeding degradation. The increases in Rα2 mRNA we observed were paralleled by concomitant increases in both intracellular and secreted Rα2 protein, most evident with PGE_2_. While the increment of protein induction was substantially less than that of mRNA, it was sufficient to be associated with blunted IL-13 induction of its key target gene, the matricellular protein periostin. These results suggest that additional regulation may exist at the levels of protein translation and/or stability. Moreover, the lack of an additive effect of PGE_2_ + FGF-2 on periostin expression could be explained either by the fact that the PGE_2_ dose employed was sufficient for maximal reduction in periostin gene expression, or that PGE_2_ and FGF-2 had no additive effect in this context. Comprehensively analyzing a potential additive effect would require that co-stimulation studies be performed with suboptimal doses of both FGF-2 and PGE_2_ and then stimulated with IL-13 to measure periostin gene expression. Evaluating these possibilities will require additional studies. Future work might also evaluate the impact of PGE_2_ and FGF-2 on IL-13 target genes other than periostin.

Studies by our laboratory and others have definitively established that the predominant signaling pathway for PGE_2_ in Fibs proceeds through the binding to EP2 with subsequent activation of adenylyl cyclase to generate cAMP and resulting activation of PKA. Consistent with an EP2/cAMP/PKA pathway being operative here as well, induction of Rα2 was mimicked by the EP2 agonist butaprost and by the receptor-independent activation of adenylyl cyclase by forskolin, while induction by PGE_2_ was completely abrogated by the PKA inhibitor PKI 14–22 amide. We and others have reported that the ability of FGF-2 to promote Fib proliferation and migration critically depends on signaling via the PI3 kinase-PDK1-AKT pathway^46^. Since Rα2 induction by FGF-2 was attenuated by inhibitors of all three of these sequential kinases, and was mimicked by expression of constitutively active AKT, we conclude that this pathway is operative in Rα2 transcription as well. PI3 kinase activation is also implicated in the mitogenic actions of PDGF, and it is therefore likely that that the PI3 kinase-PDK1-AKT pathway is required for Rα2 expression by diverse mitogens. Although the well-characterized pro-fibrotic factor TGF-β is also known to activate PI3 kinase^63^, it failed to upregulate Rα2 expression. This may reflect differential activation by distinct growth factors of PI3 kinase isoforms with varying capacities to initiate Rα2 transcription, a prospect that will require experimental evaluation.

Most mediators thus far reported to increase gene expression of Rα2 – including IL-13, TNF-α, and IL-17 – possess predominant pro-inflammatory actions. Among mediators with Fib modulatory actions, only the pro-fibrotic substance LPA has been shown to increase gene expression of Rα2^42^. Increasing expression of this decoy receptor for pro-inflammatory and pro-fibrotic IL-13 thus allows these substances to activate a homeostatic brake on pathologic responses. As mentioned earlier, intrinsic PGE_2_ effects on Fibs are largely suppressive, and the same is also the case for its effects on a variety of leukocyte subsets. Induction of Rα2 thereby serves to amplify the anti-fibrotic and anti-inflammatory actions of this lipid mediator. In contrast to PGE_2_, the effects of FGF-2 on Fibs are more complex. Its well-known mitogenic and migratory actions, noted above, promote tissue fibrosis, and indeed, FGF-2 has been implicated in the pathogenesis of IPF^64^. On the other hand, FGF-2 has also been reported to possess anti-fibrotic actions in certain contexts^65^. Induction of Rα2 may represent a previously unrecognized mechanism limiting the fibrogenic actions of FGF-2. We have previously demonstrated that PGE_2_ strongly blocks the proliferative^46^ and migratory^49^ actions of FGF-2 in Fibs. For this reason, it was unexpected that these two mediators would exert parallel stimulatory effects on Rα2 transcription. That PGE_2_ and FGF-2 would act synergistically to enhance Rα2 transcription was even more surprising. This motivated us to explore the mechanisms responsible for synergistic induction by these substances.

We confirmed findings reported by others^43^ that phosphorylation and activation of STAT6 accompanied IL-13-induced Rα2 transcription, and a STAT6 inhibitor abrogated such induction. By contrast, STAT6 activation was not observed with FGF-2 or PGE_2_, and the induction of Rα2 by these mediators was unaffected by a STAT6 inhibitor, implicating STAT6-independent mechanism(s) in Rα2 induction. A STAT6-independent mechanism for Rα2 induction has been reported previously for TNF-α, though the mechanism was not elucidated^14^. As a strategy to identify potential transcription factors other than STAT6 responsible for Rα2 gene induction, we performed transcription factor binding site analysis using the transcription factor database MatInspector. This revealed the presence of numerous CREB binding sites within the Rα2 promoter region. To our knowledge, the role of CREB in Rα2 gene regulation has not previously been investigated. Its functional capacity to influence Rα2 expression in Fibs was first confirmed by expressing a constitutively active form of CREB in lung Fibs. Phosphorylation and activation of CREB has been previously reported in response to both PGE_2_ and FGF-2^50–53^, and we confirmed that both of these mediators increased the phosphorylation of CREB in Fibs. The ability of a potent and selective CREB inhibitor to abrogate PGE_2_- and FGF-2-induced Rα2 expression provided the critical link between the activation of this transcription factor and its functional role in receptor gene expression. In addition to CREB binding sites, recent studies revealed binding sites for activator protein-1 (AP-1) in the Rα2 promoter region^66^. Of note, prior studies implicated a role for AP-1 in FGF-2-mediated gene regulation^67,68^. It is thus possible that CREB and AP-1 may act cooperatively in FGF-2-driven Rα2 gene expression, and future studies will be needed to address such a possibility.

The fact that both PGE_2_ and FGF-2 acted through CREB made their striking synergy in Rα2 induction even more curious. The STAT6 binding sites implicated in IL-13-induced Rα2 expression are in close proximity to TSS1. On the other hand, the CREB binding sites are positioned close to both TSS1 and TSS2. Experiments using TSS-specific qPCR primers further confirmed that TSS1 was utilized for Rα2 transcription initiated by IL-13. TSS1 was also shown to be the major start site for transcription initiated by PGE_2,_ whereas TSS2 was exclusively used for transcription initiated by FGF-2. CREB-mediated Rα2 transcription elicited by the combination of an agonist utilizing TSS2 (FGF-2) along with an agonist utilizing TSS1 (either PGE_2_ or IL-13) was accompanied by activation of both TSSs, likely explaining the synergistic effects observed for Rα2 expression. The relationship between individual transcription factor binding sites and transcription initiated at the two TSSs remains uncertain. The activation of transcription from both TSS1 and TSS2 also provides a potential explanation for synergistic patterns of induction by various combinations of stimuli. In this regard, it is of interest to note that synergistic induction of Rα2 in lung Fibs was previously observed with the combination of IL-17 and either IL-13 or TNF-α^69^. As the mechanism underlying such synergy was never explored, it will be of interest in future studies to test the relevance of the dual TSS mechanism with these stimuli as well. Similarly, the possibility of functional differences between TSS1- and TSS2-initiated Rα2 transcripts will require further investigation.

Rα2 is unique among IL-13 receptors in that its expression is dynamically regulated and is known to be either increased or decreased in various disease states. Nevertheless, understanding of the mechanisms governing its transcriptional regulation has been limited. Our study expands the range of mediators capable of robustly stimulating Rα2 transcription to include mitogens as well as PGE_2_. We also identify CREB as a new transcriptional regulator for Rα2. Finally, we provide evidence for dual TSS utilization as a possible mechanism underlying synergy in Rα2 transcriptional activation. Future studies will be needed to evaluate the relevance of these new mechanistic insights to other transcriptional regulators of Rα2, alone and in combination, and to alterations in its expression in various pathologic states.

## Materials and Methods

### Cell culture and reagents

CCL210 (CCD-19Lu) primary Fibs isolated from normal adult human lung, A549 human lung adenocarcinoma cells, Beas-2b bronchial epithelial cells, and MLE-12 murine lung epithelial cells were purchased from ATCC. Fibs were cultured in low glucose Dulbecco’s modified Eagle’s medium and epithelial cells were cultured in RPMI-1640 medium (both purchased from Invitrogen) and supplemented with 10% fetal bovine serum (Hyclone), 100 units/ml penicillin (Gibco), and 100 μg/ml streptomycin (Invitrogen). Recombinant human IL-13, FGF-2, PDGF, TNF-α, and TGF-β, as well as LPA, were purchased from Millipore. PGE_2_, forskolin, butaprost, Act D, LPA, PI3 kinase inhibitor (LY294002) and AKT inhibitor (triciribine) were purchased from Cayman Chemicals. GSK 2334470 was from Tocris Bioscience. PKI 14–22 amide and CREB inhibitor 666–15 were purchased from Millipore Sigma. STAT6 inhibitor AS1517499 was purchased from Axon Medchem. Unless otherwise specified, the final concentrations of agonists used for cell treatment were: TGF-β (2 ng/ml), FGF-2 (50 ng/ml), PDGF (50 ng/ml), IL-13 (10 ng/ml), TNF-α (10 ng/ml), LPA (10 μM), PGE_2_ (500 nM), forskolin (10 μM), butaprost (10 μM), PKI (10 μM), LY294002 (10 μM), GSK 2334470 (1 μM), triciribine (5 μM), 666–15 (500 nM), AS1517499 (250 nM) and Act D (2 μg/ml). Antibody recognizing Rα2 was purchased from Abcam. Antibodies recognizing CREB, pCREB, STAT6, pSTAT6, and GAPDH HRP conjugate were purchased from Cell Signaling Technologies. High capacity cDNA reverse transcription kit and Fast SYBR Green Master Mix were from Applied Biosystems. Dual-Luciferase Reporter Assay reagents were purchased from Promega.

### Harvesting Fib culture supernatant

Supernatant from Fib cultures was collected after 48 h culture. Supernatant was then sequentially centrifuged at 500 × *g* for 10 min and 2500 × *g* for 12 min to remove dead cells/debris and apoptotic bodies, respectively. Equal volumes of supernatants from each culture were then concentrated 50-fold using Amicon Ultra-10 centrifugal filters (Millipore) and immediately subjected to western blotting to detect Rα2 protein.

### Acquisition of human Fibs, alv mϕs, and AEC2s

From the University of Michigan lung tissue biorepository, we obtained IRB-exempted primary type II alveolar epithelial cells and primary lung Fibs from lungs of several subjects lacking lung pathology. Likewise, primary alv mϕs were purified from bronchoalveolar lavage samples obtained from subjects undergoing research bronchoscopy at the University of Michigan Hospital Medical Procedure Unit. Subject samples utilized in this study included two with asthma and one non-asthmatic atopic individual; since no differences were noted among these subjects in alv mϕ expression of Rα1 or Rα2, they were analyzed as a single group. Informed consent was obtained from each subject prior to sample collection in accordance with the Declaration of Helsinki and with approval of the Institutional Review Board (UM IRB# HUM00136068). Lavage fluid samples were subjected to centrifugation at 500 × *g* for 10 min (4 °C), and pelleted cells were resuspended in complete RPMI 1640 medium (containing fetal bovine serum and other supplements described in Cell Culture and Reagents) and cultured overnight at a density of 0.6 × 10^6^ cells/mL. Non-adherent and loosely adherent cells were washed off with PBS, and the remaining cultures of adherent cells were > 98% alv mϕs by Diff-Quik staining.

### Isolation of murine lung Fibs and alv mϕs

Pathogen-free naive male C57BL/6 mice aged 6–8 weeks were purchased from The Jackson Laboratory. Mice were housed in groups of 5 and they had *ad libitum* access to water and food. All methods were carried out in accordance with relevant national and local guidelines and regulations regarding the use of experimental animals and with approval of the University of Michigan Committee for the Use and Care of Animals. Mice were sacrificed and lung lavage and alv mϕ isolation and culture were performed as described previously^70^. Fibs were also outgrown from lung tissue and cultured as described previously^46^.

### RNA isolation and quantitative real-time PCR

Cells were suspended in 700 μl TRIzol reagent (ThermoFischer Scientific) and RNA was extracted using the RNeasy Mini Kit (Qiagen) according to the manufacturer’s instructions. The concentration of total RNA was measured using Nanodrop. Using the high capacity cDNA reverse transcription kit (Applied Biosystems), total RNA was converted to cDNA. Levels of mRNA were assessed by quantitative real-time PCR (qPCR) analysis with a Fast SYBR green master mix (Applied Biosystems) on an ABI Prism 7300 thermocycler (Applied Biosystems). Expression of human Rα1, Rα2, TSS1- and TSS2-specific Rα2, and periostin and murine Rα1 and Rα2 was assessed using sequence-specific primers listed in Supplementary Table 2. Unless specified otherwise, the human Rα2 primer employed was designed to bind downstream of exon 3 and is common to both TSS1- and TSS2-initiated transcripts. Relative gene expression was determined by the ΔCT method, and GAPDH and β-actin were used as a reference gene for human and mouse samples, respectively.

### Rα2 promoter activity assay

The Rα2 promoter-luciferase construct (pGL3-Rα2) was a kind gift from Dr. Wei Xu (McArdle Laboratory for Cancer Research, University of Wisconsin-Madison, Madison, WI)^71^. Cells were grown on 6-well plates and co-transfected at 60% confluence with FuGENE HD (Promega) using 1.0 μg of pGL3-Rα2 or empty (pGL3-Basic) plasmids together with 50 ng of a reference promoter driving Renilla luciferase (pRL-TK) to normalize the data. After 24 h of incubation, cells were stimulated ± PGE_2_, FGF-2, or IL-13 for an additional 24 h. Cells were then lysed and firefly and Renilla luciferase activities were measured by the Dual-Luciferase reporter assay system using a GloMax 96 microplate luminometer with dual injectors (Promega). Results were normalized by dividing the firefly luciferase activity by the Renilla luciferase activity of the same sample as described previously^48^.

### Plasmid overexpression studies

The Myr-AKT construct was kindly provided by Dr. Philip Tsichlis (Tufts University, Boston, Massachusetts, USA) and the active CREB construct (pCREB-VP16) was a generous gift from Dr. Angel Barco (Instituto de Neurociencias, Universidad Miguel Hernández, Spain)^72,73^. Untagged human Rα2 overexpression vector (pCMV6-Rα2) and control vector (pCMV6-empty) were purchased from OriGene. Transient transfection studies were carried out using Fugene HD (Promega). Briefly, plasmid and transfection reagents were mixed at a 1:3 DNA/Fugene HD ratio in Opti-MEM reduced serum medium (Invitrogen), incubated for 20 min at room temperature, and then added to the cells. After 24 h after transfection, culture media was changed to serum-free medium and stimulated and cells were harvested per the experimental protocol described under Results section.

### Western blot analysis

Samples were lysed in RIPA buffer (Cell Signaling) supplemented with protease inhibitors (Roche Diagnostics) and phosphatase inhibitor cocktail (EMD Biosciences). Samples were analyzed as previously described^74^. All antibodies were used at a dilution of 1:1,000 except GAPDH-HRP which was used at a dilution of 1:5000.

### SspI restriction digestion

Using TSS-specific primers (listed in Supplementary Table 2C), TSS1 and TSS2 products were amplified by PCR. By running in 1.5% agarose gel, TSS1- and TSS2-products were confirmed, gel extracted, and 500 ng of DNA was digested with Ssp1 enzyme (from NEB) treatment overnight. Samples were compared with undigested DNA using 2% agarose gel.

### TSS-specific Rα2 amplification

To PCR amplify the TSS-specific Rα2 transcripts listed in Supplementary Table 2C, we employed TSS-specific forward primers with a common reverse primer that binds to Rα2 on exon 10 (see Fig. 8B). PCR products were analyzed by running the samples in 1.0% agarose gel. DNA bands were then excised from the gel, purified using Qiagen Gel Extraction kit and samples were subjected to DNA Sanger sequencing.

### Statistical analysis

Data are presented as means and were analyzed for statistical significance by one-way ANOVA and Dunnett’s multiple comparisons post-hoc test using Prism 7.0 (GraphPad Software). Error bars represent mean values (±S.E.). p-values below 0.05 were considered to be statistically significant.

## Supplementary information

Supplementary information.
