# Supplementary material for: Transcriptional regulation of the IL-13Rα2 gene in human lung fibroblasts
Source: Sci Rep. 2020 Jan 23;10:1083. doi: 10.1038/s41598-020-57972-1 (PMC6978327; doi:10.1038/s41598-020-57972-1)
Supplement: Supplementary file 1 — Supplementary information. [file 41598_2020_57972_MOESM1_ESM.pdf]

Supplementary Information

Transcriptional regulation of the IL13Rα2 gene in human lung fibroblasts

Loka R. Penke[1], Hideyasu Ouchi[1], Jennifer M. Speth[1], Yvonne J. Huang[1], and Marc Peters-Golden[1, 2, *]

[1]Division of Pulmonary and Critical Care Medicine
Department of Internal Medicine
University of Michigan Medical School
Ann Arbor, MI, USA

[2]Graduate Program in Immunology

[*]Corresponding author:

Marc Peters-Golden, M.D.
Phone: 734-936-5047
Email: petersm@umich.edu

Supplementary Table 1. Delta Ct values for human Rα1 and Rα2 (normalized to GAPDH).

	Delta Ct values for Rα1		Delta Ct values for Rα1	
	Ct1	Ct2	Ct1	Ct2
CCL210 1	0.413115	-0.41311	9.82E-05	-9.8E-05
CCL210 2	-0.00504	0.005037	-1.55308	-1.61515
CCL210 3	-0.03496	-0.25778	-0.89437	-0.91602
Fib 1	-0.75432	-0.83912	0.024089	-0.13572
Fib 2	0.49419	0.483061	-0.13533	-0.12415
alv mΦ1	-3.69043	-3.7052	10.84315	5.48309
alv mΦ2	-3.07409	-3.25203	6.935205	7.28216
alv mΦ3	-3.71732	-3.74306	5.699548	6.854309
AEC2 1	-3.049	-3.0001	2.578239	2.882716
AEC2 2	-4.03311	-3.89369	3.287333	3.553622
AEC2 3	-3.32917	-3.22623	3.742526	3.361178
AEC2 4	-4.16697	-4.08801	3.271862	3.059289
A549	-1.18881	-1.21936	10.66169	10.61898
Beas-2b	0.062138	0.020191	6.825636	6.838631

Supplementary Table 2. List of primer sequences.

Gene	Forward sequence	Reverse sequence	Amplicon length (in base pairs)
**A. Primers for qPCR amplification**			
hRα1	AGGAATACCAGTCCCGACAC	TGGAATCCTTCACTTTGGTC	139
hRα2	TCTTGGAAACCTGGCATAGG	TGCCTCCAAATAGGGAAATC	147
GAPDH	CAGCCTCAAGATCATCAGCA	ACAGTCTTCTGGGTGGCAGT	138
c-FOS	CCGAAGGGAAAGGAATAAGA	CTTCTCCTTCAGCAGGTTGG	145
POSTN	GCAGAGAAATCCCTCCATGA	TGTCCAGTCTCCAGGTTGTG	114
(m) beta actin	GACGGCCAGGTCATCACTAT	GCACTGTGTTGGCATAGAGG	166
mIL13rα1	CACCATTCCAGTCTTTGTCG	TCCAGTGCAGGGTATCATCA	149
mIL13rα2	GAAGGTTACACAGGGCCAGA	GAGGCTCAATGTGGGTTCAG	126
**B. TSS-specific primers for PCR amplification (used in Figure 8C)**			
TSS1-specific Fwd	CAATCCTGTAACCCAGAAGCA	CCAAATGGTAGCCAGAAACG (designated as Rα2_Rev1)	1320
		GGGTAGGTGTTTGGCTTACG (designated as Rα2_Rev2)	1383
TSS2-specific Fwd	GCCATTGAGGCTTACCAAAG	CCAAATGGTAGCCAGAAACG (designated as Rα2_Rev1)	1265
		GGGTAGGTGTTTGGCTTACG (designated as Rα2_Rev2)	1328
**C. TSS-specific primers for qPCR amplification (used in Figure 8E)**			
TSS1-specific Fwd	CAATCCTGTAACCCAGAAGCA (designated as Fwd Primer1)	TCTCCATAGCAACCGTCTCC (designated as Rev Primer1)	72
TSS2-specific Fwd	GCCATTGAGGCTTACCAAAG (designated as Fwd Primer2)		64
**D. TSS-specific primers for regular PCR amplification (used in Supplementary Figure 5)**			
TSS1-specific Fwd	CAATCCTGTAACCCAGAAGCA (designated as Fwd Primer1)	TCTCGGTGTCTGAAGATGAAG (designated as Rev Primer2)	315
TSS2-specific Fwd	GCCATTGAGGCTTACCAAAG (designated as Fwd Primer2)		307

Supplementary Figure legends

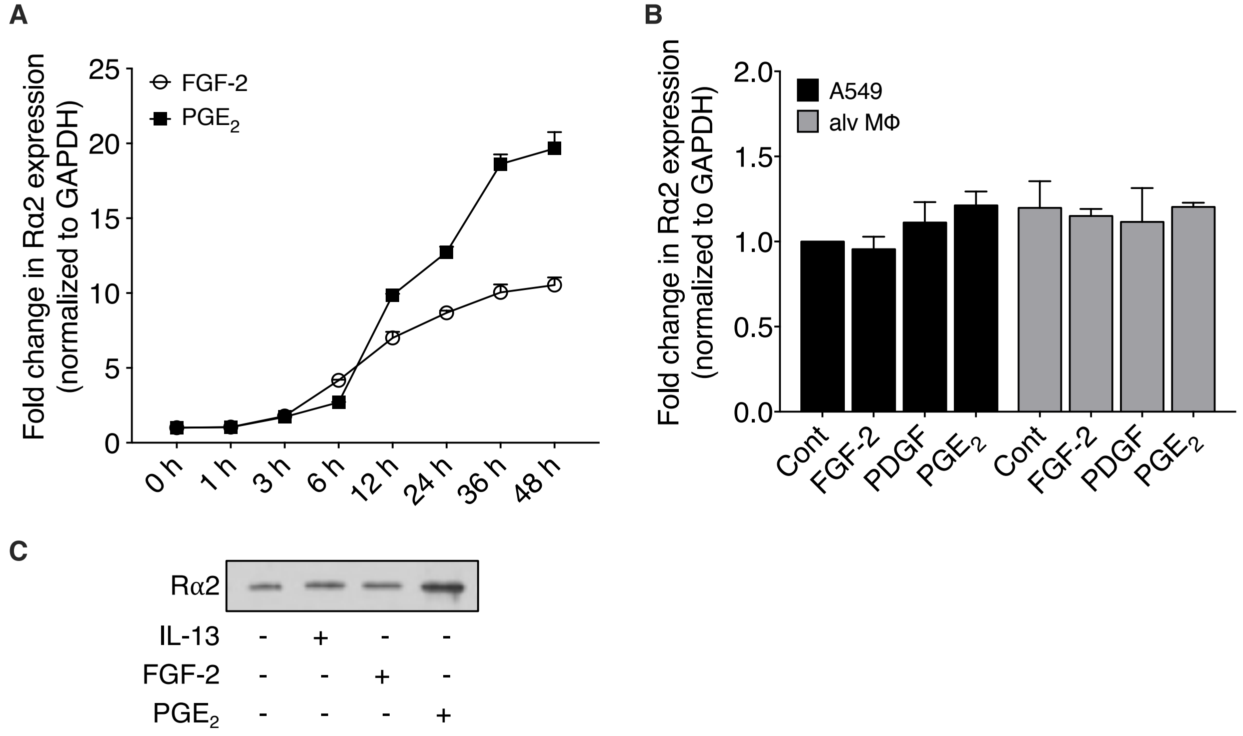

Supplementary Figure 1. Induced expression of Rα2 in various lung cell types. (**A**) qPCR analysis of PGE_2- and FGF-2-induced Rα2 expression kinetics and Rα2 in human CCL210 lung fibroblasts. (**B**) A549 and primary alv mϕs were stimulated with FGF-2, PDGF, or PGE_2 for 24 h and expression of Rα2 was determined using qPCR analysis. (**C**) CCL210 lung fibroblasts were stimulated with IL-13, FGF-2, or PGE_2 and release/secretion of Rα2 protein into the culture supernatant was assessed by western blot analysis of concentrates of equal volumes of supernatants. Expression is relative to that of untreated sample. Each bar represents mean values (± S.E.) from three independent samples. *$p<0.05$.

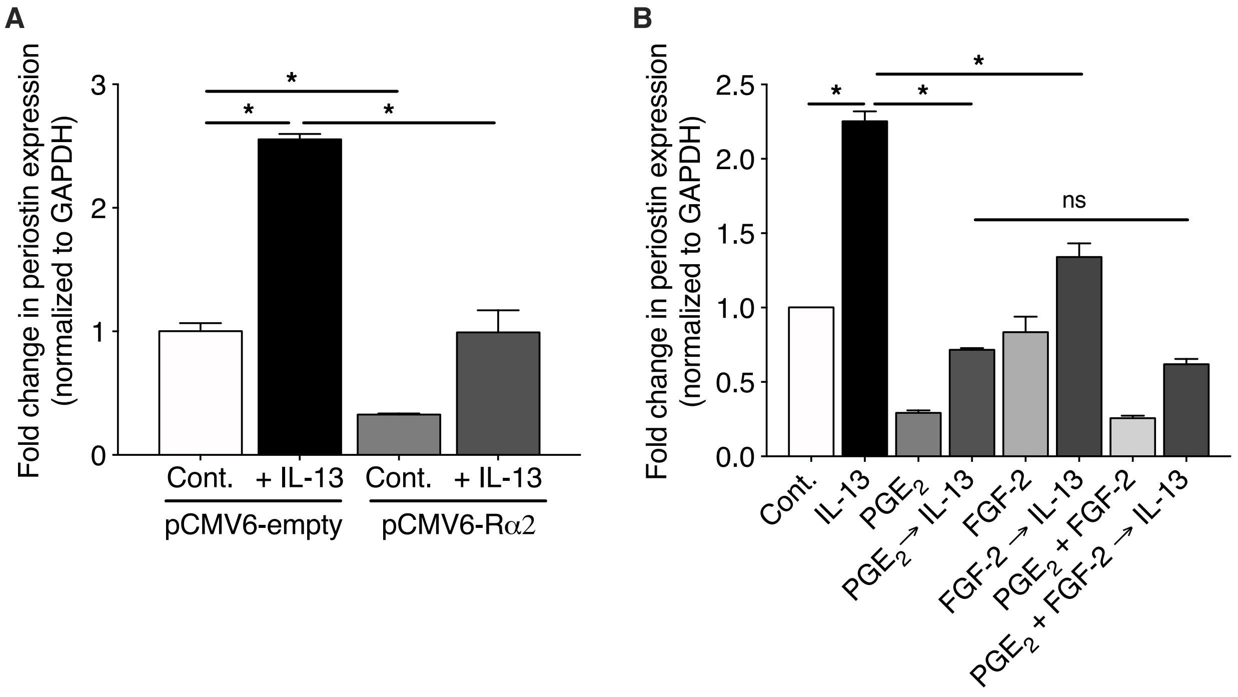

Supplementary Figure 2. Inhibition of IL-13-induced periostin gene expression by forced overexpression of Rα2, or by stimulation with PGE_2 or FGF-2 in human lung fibroblasts. (A) CCL210 cells were transfected with pCMV6 (empty) vector or Rα2 overexpression construct (pCMV6-Rα2). 24 h post transfection, cells were stimulated ± IL-13 for additional 24 h, and the expression of periostin was determined using qPCR analysis. (B) CCL210 cells were pre-treated ± FGF-2 or ± PGE_2 or both (i.e., FGF-2 + PGE_2) for 24 h followed by stimulation ± IL-13 for addition 24 h, and the expression of periostin was determined using qPCR analysis. Each bar represents mean values (± S.E.) from three independent experiments. * $p<0.05$; ns, not significant.

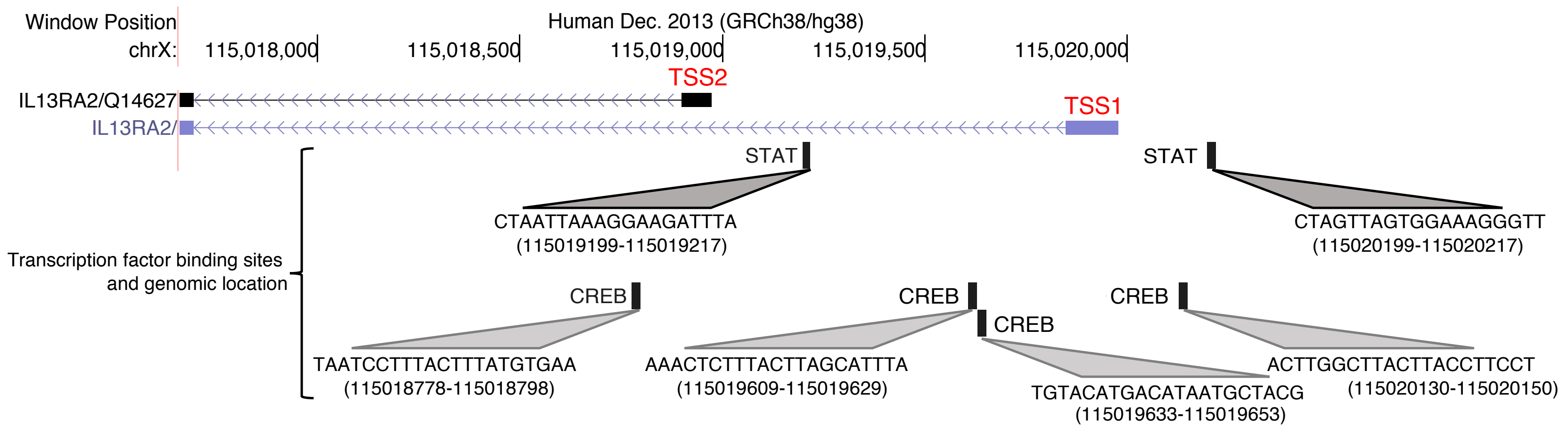

Supplementary Figure 3. STAT and CREB binding sites in the proximity of Rα2 TSSs. Diagram depicts a MatInspector-based analysis of potential STAT and CREB binding sites within the Rα2 promoter region spanning the two TSSs of interest.

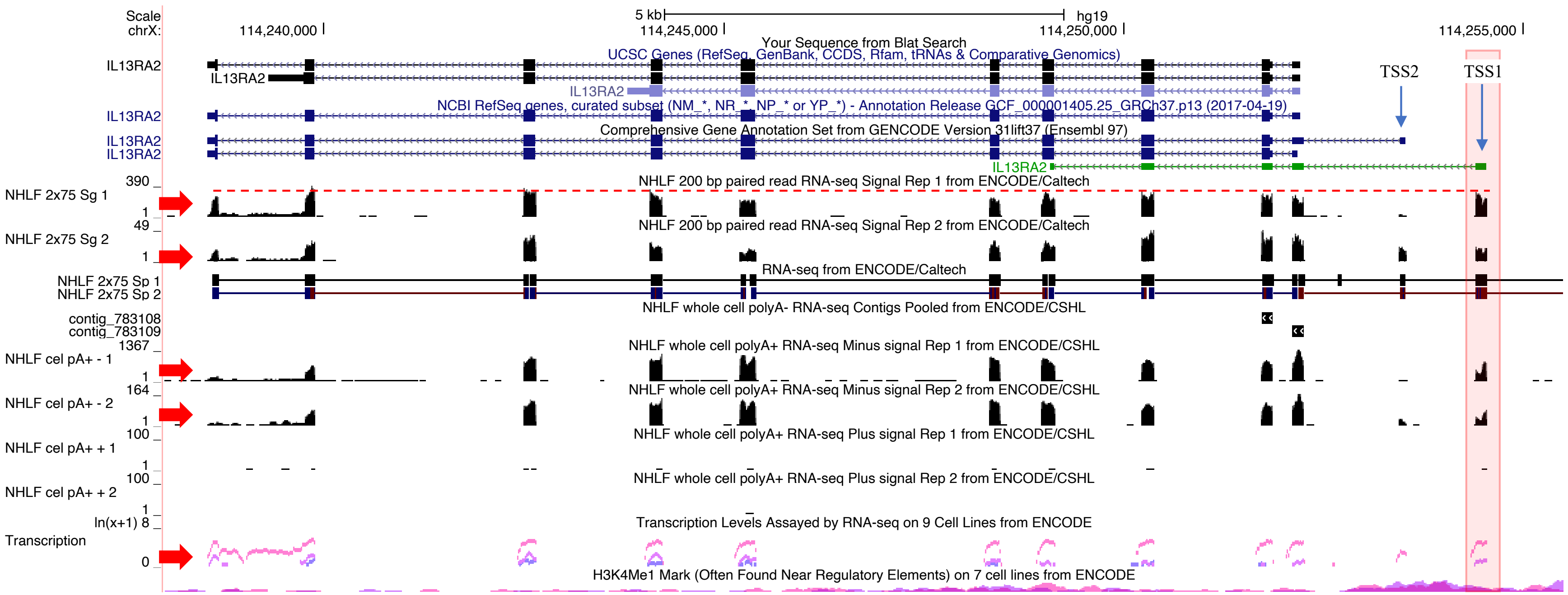

Supplementary Figure 4. RNA-seq data from normal human lung Fibs showing utilization of alternative transcription start sites. Diagram depicts polyA+ RNA-seq data from UCSC genome browser with expressed exons as reflected by the peak height (shown in red arrow), TSS1 and TSS2 were identified with down arrows (blue), and the highlighted vertical bar to point the TSS1-specific exon peak which is comparable across the gene (shown with dotted red line).

A

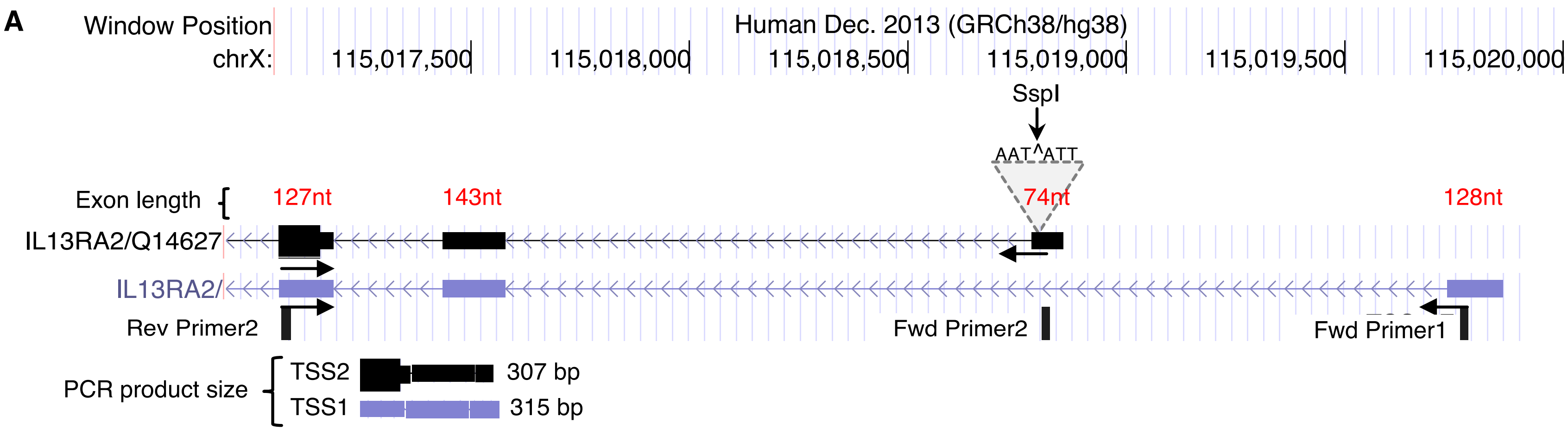

B

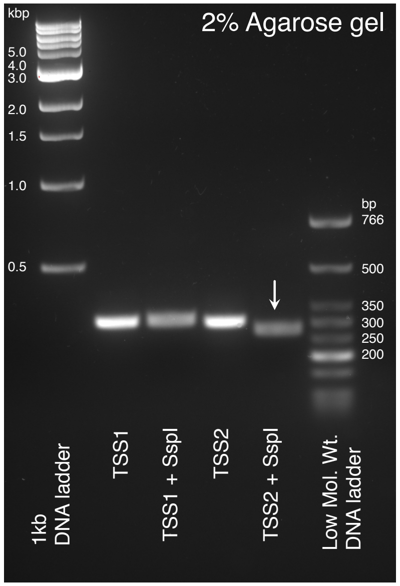

- PCR product from TSS1-specific Fwd primer:
CAATCCTGTAACCCAGAAGCAaaggagagaattgtctttgtgttcatttgggggagacggttgctatggagatggatgatatcata
actccattgtgaaccagtaagaacactctcgtgagtctaacggtcttccggatgaaggctatttgaagtcgccataacctggtcagaagtgtgc
ctgtcggcggggagagaggcaatatcaaggttttaaatctcggagaaatggctttcgtttgcttggctatcggatgcttatatacctttctgataag
cacaacatttggctgtaCTTCATCTTCAGACACCGAGA

- PCR product from TSS2-specific Fwd primer:
GCCATTGAGGCTTACCAAAGttacaat^attacagatggatcaggggagacggttgctatggagatggatgatatcataactcca
ttgtgaaccagtaagaacactctcgtgagtctaacggtcttccggatgaaggctatttgaagtcgc
cataacctggtcagaagtgtgcctgtcggcggggagagaggcaatatcaaggttttaaatctcggagaaatggctttcgtttgcttggctatcgg
atgcttatatacctttctgataagcacaacatttggctgtaCTTCATCTTCAGACACCGAGA

- SspI digests TSS2 PCR amplicon into 280bp and 27bp.

Supplementary Figure 5. Characterization of TSS transcripts. (A) Diagram depicts PCR primer binding sites on Rα2 exons for TSS-specific amplification and SspI restriction site sequence (AAT^ATT, designated by underline) within the TSS2. (B) Agarose gel image showing PCR-amplified products of TSS1 and TSS2, depicting cleavage of TSS2 by SspI enzyme (left). Nucleotide sequence of TSS-specific amplicons and digested product lengths of TSS2 after SspI cleavage (right).

.
